# An Analysis of Psychiatric Workforce Distribution in the Philippines

**DOI:** 10.3390/healthcare14081064

**Published:** 2026-04-17

**Authors:** Joseph P. Anlacan, Veeda Michelle M. Anlacan, Harold Joshua D. de Guzman, Beatrice M. Anlacan, Roland Dominic G. Jamora

**Affiliations:** 1 Department of Psychiatry and Behavioral Medicine, College of Medicine, Philippine General Hospital, University of the Philippines Manila, Manila 1000, Philippines; 2 Department of Neurosciences, College of Medicine, Philippine General Hospital, University of the Philippines Manila, Manila 1000, Philippines; vmanlacan@up.edu.ph (V.M.M.A.); rgjamora@up.edu.ph (R.D.G.J.); 3 Institute on Aging, National Institutes of Health, University of the Philippines Manila, Manila 1000, Philippines; 4 Office of the Vice Chancellor for Research, University of the Philippines Manila, Manila 1000, Philippines; hddeguzman@up.edu.ph; 5 College of Medicine, University of the Philippines Manila, Manila 1000, Philippines; bmanlacan@up.edu.ph

**Keywords:** universal health care, psychiatry, regional distribution, psychiatrist, Philippines, population, workforce

## Abstract

**Background:** In the Philippines, studies have shown that availability and access to healthcare varies widely. Although the shortage of psychiatrists in the country has been recognized for many years, no published study to date has described their distribution across the regions. This study aimed to describe the distribution of psychiatrists in the country using publicly available data on the Internet. **Methods:** This was a cross-sectional study, analyzing publicly available data from the Philippine Psychiatric Association (PPA) web directory, the Philippine Health Insurance Corporation (PhilHealth) web database of accredited psychiatrists, and the Philippine Statistics Authority. Information on location of practice, sex, PPA membership, PhilHealth accreditation, regional gross domestic product (GDP), and regional population were collated. **Results:** Information on 409 psychiatrists was available online, with 68% being female and 53% holding PhilHealth accreditation. There were a total of 417 declared locations of practice, with six psychiatrists practicing in more than one location. The National Capital Region accounted for 53.5% of the declared practice locations, while no psychiatrist declared practicing in the Bangsamoro region. **Conclusions:** This study highlights the maldistribution of psychiatrists across the Philippines. Policies to incentivize and encourage practice in low-access regions and investment in technology, such as telemedicine, may help reduce the access gap.

## 1. Introduction

Universal health care (UHC) is “the provision to every Filipino of the highest possible quality of health care that is accessible, efficient, equitably distributed, adequately funded, fairly financed, and appropriately used by an informed and empowered public” [1]. A consequential necessity for achieving equitable distribution would be the optimization of spatial accessibility. Guagliardo [2] identifies two dimensions of spatial accessibility, availability and accessibility. Availability refers to the number of local service points open to a patient, and accessibility refers to the travel impedance between patient location and service points. Patients are thus disadvantaged where services are unavailable and/or inaccessible.

A recent study in 2024 investigated the spatial accessibility of outpatient and inpatient services in the Philippines [3]. They found that the northwestern region of the country in general had more spatial accessibility compared to the underserved central and southern regions. Metropolitan areas had more spatial accessibility for inpatient services, while rural and mountainous regions had more spatial accessibility for outpatient services. Some select rural areas were even found to be devoid of local healthcare services. However, service delivery spots were not delineated by quality, level, or scope of service provision: a hospital may be geographically accessible but lack professionals with the necessary expertise for specific cases, such as psychiatric conditions.

Psychiatric services can be broadly defined as specialized healthcare services aimed at the diagnosis, treatment, and management of mental, emotional, and behavioral disorders as part of holistic medical care. Psychiatric services constitute both medical interventions (i.e., prescription medicines), and non-medical interventions (i.e., psychotherapy), whereas psychological services are exclusively non-medical interventions. Different roles exist in the provision of these services.

Exact data on the distribution of psychiatric service providers in the Philippines is not available. However, it can be hypothesized that they are concentrated in regions with high spatial accessibility for inpatient services, as mental health (MH) care in the country has been reported to be primarily delivered in these areas [4,5]. Additionally, the existence of different types of MH professionals may complicate the interpretation of availability data.

Republic Act No. 11036 defines a MH professional as “a medical doctor, psychologist, nurse, social worker or any other appropriately trained and qualified person” skilled in providing MH services [6]. The American Psychological Association [7] further delineates these roles: social workers provide counseling and connect patients to their communities. Psychologists diagnose and provide psychotherapy but cannot prescribe drugs or order tests. Finally, psychiatrists can perform the functions of both, and as doctors, have legal authority to prescribe medications, request tests, and order interventions such as admissions [8]. Consequently, areas without psychiatrists cannot effectively manage psychiatric patients requiring comprehensive medical interventions.

Moreover, MH professional availability is compromised by significant shortage. The WHO Special Initiative on Mental Health in 2020 noted that the Philippines has an estimated 548 psychiatrists (0.5 per 100,000 population), 516 psychiatric nurses (0.5 per 100,000 population), and 133 psychologists (0.1 per 100,000 population) [9]. Overall, the fragmented landscape of MH service delivery fails to adequately match the prevalence of MH disorders.

Studies have attempted to determine MH disorder prevalence across varying scopes. A community-based study in Barangay Tenejero, City of Balanga, Bataan, found that among 419 participants, 109 (26.01%) experienced at least one core symptom of a psychiatric disorder while 44 (10.5%) had at least one psychiatric disorder, with major depressive disorder (3.58%) being the most common, followed by psychotic disorders (1.91%) [10].

**Figure 1:**
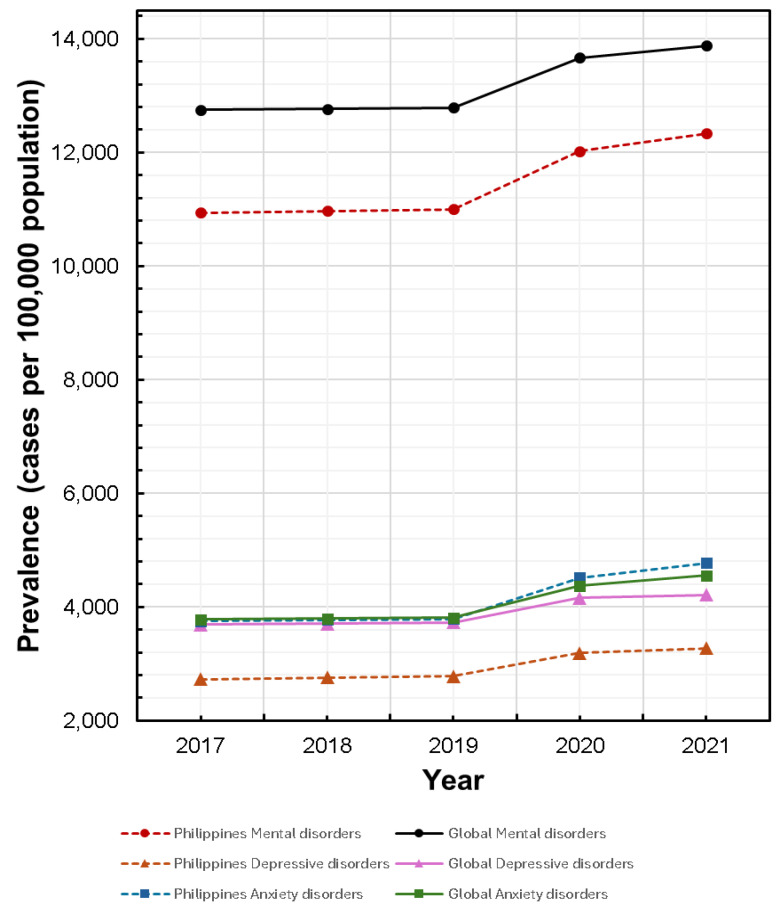
Estimated prevalence of MH disorders globally and in the Philippines (adapted from the 2021 global burden of disease study).

However, cross-sectional studies capture only a specific point in time, and with MH disorders being chronic diseases, data on prevalence over time is missed. Figure 1 shows prevalence estimates from 2017 to 2021 based on the latest Global Burden of Disease study [11,12], which suggests a rising trend both globally and locally. For mental health disorders, the global prevalence rate was estimated at 12,742 cases per 100,000 population in 2016, which rose to 13,880 cases per 100,000 population in 2021. In the Philippines, the prevalence rate was estimated to be 10,906 cases per 100,000 population in 2016, which rose to 12,336 per 100,000 population in 2021.

Beyond their immediate effect, many MH disorders contribute to disability [12,13], translating to monetary and non-monetary costs [14,15]. Thus, MH disorders constitute a significant burden on the patient, the family and the community.

Depending on the case of MH disorder, intervention may require a combination of medical and non-medical interventions. Thus, psychiatric services are necessary because they can provide medical interventions that cannot be rendered by other MH providers.

Therefore, the number and distribution of accessible psychiatrists is crucial to determine if the burden is adequately being addressed. As a result, this study aimed to determine geographical psychiatrist distribution, analyze descriptive data, and identify underserved areas. This information may help guide policymakers in creating policies and programs that target areas with poor resource.

## 2. Methods

### 2.1. Study Design

This was a cross-sectional study analyzing publicly available data on psychiatrists in the Philippines. The following data sources were used: (1) Philippine Psychiatric Association (PPA) web directory of board-certified psychiatrists [16], (2) PhilHealth web database of accredited psychiatrists [17], and (3) Philippine Statistics Authority (PSA) mid-year population data [18].

We also included the gross regional domestic product (GRDP) and the number of training institutions to assess potential associations using the PSA 2022 to 2024 GRDP (base year 2018) [19], and the PPA online list of accredited training institutions [20].

Ethics approval was not sought as the study did not involve human subjects or patient data.

### 2.2. Inclusion/Exclusion Criteria

Data on board-certified psychiatrists practicing in the Philippines was included. Data on three psychiatrists listed on the PPA website but not actively practicing in the country were excluded.

### 2.3. Data Collection

A list of psychiatrists was collated from the publicly available list on the PPA website [16]. The following information was gathered: location of practice, PPA membership type, and PhilHealth accreditation status. Sex of psychiatrist was not stated on the PPA website but was assumed based on the name of the psychiatrist. That is, psychiatrists with traditionally male names were listed as male, while psychiatrists with traditionally female names were listed as female.

### 2.4. Data Analysis

Data was analyzed using descriptive statistics in Microsoft Excel [21]. Geographic data was visualized via choropleth mapping using Quantum Geographic Information System version 3.42.0 [22].

Analyses were selected post hoc based on visual inspection of the data and the goals of exploratory pattern identification. Exploratory statistical analyses were performed in R version 4.5.1 [23]. Poisson regression was initially conducted to explore relationships between the number of psychiatrists per region and selected regional indicators (population, GDP, and number of training institutions). As overdispersion was detected in the Poisson model, a quasi-Poisson regression model was subsequently created to account for this. Due to the presence of an extreme value on inspection of the data, Spearman’s rank-order correlation was computed as a nonparametric measure of monotonic association less influenced by extreme values. Given the exploratory nature of the study and the limited number of regions (n = 18), formal assumption testing was not conducted.

There was a temporal mismatch between the data sources: psychiatrist counts were from February 2025, GRDP data from 2024, and population figures from the 2020 national census. Analyses were conducted under the assumption that, despite this mismatch, the substantial difference in scale between psychiatrist counts (in tens to hundreds) and regional population or economic measures (in millions) would limit the impact of temporal variation on observed correlations.

Additionally, administrative regional boundaries changed between 2020 and 2025 due to the establishment of the Negros Island Region (NIR) in 2024. To ensure consistency specifically for the comparison between psychiatrist counts and the 2020 census population data, psychiatrists’ reported regions of practice (2025) were reclassified to align with the 2020 administrative boundaries. This reclassification was not applied to analyses involving other variables (e.g., GRDP, number of training institutions), which used the current (2025) regional configuration.

Finally, chi-squared tests of independence were used to assess associations between a psychiatrist’s declared region of practice and categorical attributes, namely PhilHealth accreditation and PPA membership type. Due to low or zero cell counts violating test assumptions, a Monte Carlo simulation with 100,000 replicates was used to obtain more reliable *p*-values. The Bangsamoro Autonomous Region, with no declared practicing psychiatrist, was excluded for having a zero-margin column.

## 3. Results

### 3.1. Descriptive Report

Based on the PPA psychiatrist directory and the PhilHealth list of accredited psychiatrists, there were a total of 409 psychiatrists in the country at the time of writing. Among these, six psychiatrists declared more than one location of practice: four had declared a practice in two locations, i.e., National Capital Region (NCR) and an additional province, while two had declared a practice in three locations, i.e., NCR and two additional provinces. Thus, the total number of declared locations of practice was 417.

**Figure 2:**
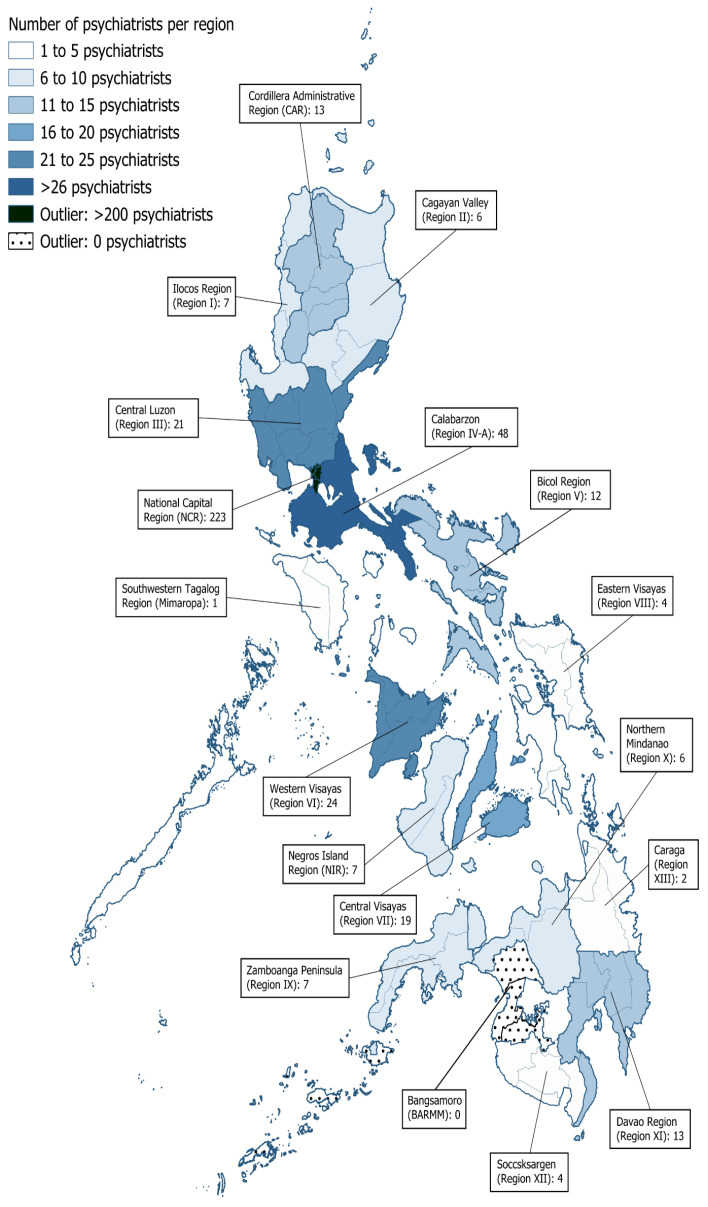
Number of available psychiatrists per region of the Philippines. Each number reflects the number of declared psychiatrists practicing in each region, with a psychiatrist being able to declare more than one region as their place of practice.

Most notably, the NCR was the location of practice of 223 out of 409 psychiatrists (54.52%) and accounted for 53.48% of the 417 total region declarations. Calabarzon had the next highest concentration of psychiatrists at 48 (representing 11.51% of all psychiatrists). In contrast, no psychiatrist declared practicing in the Bangsamoro region. The distribution of declared locations of practice is shown in Figure 2.

The ratio of psychiatrist to population per region is presented in Table 1. Luzon has the greatest number of psychiatrists (n = 331 or 1 psychiatrist: 187,906 population), followed by Visayas (n = 54; 1:381,183), and lastly by Mindanao (n = 32; 1:820,389). Accounting for the whole country, the ratio is 1 psychiatrist: 262,099 or 0.38 psychiatrist per 100,000 population.

The PPA online directory had a total of 373 entries; however, 27 were duplicate entries, and three declared practice in foreign countries. Hence, only 343 unique psychiatrists were gathered from the PPA directory. The PPA further categorizes these psychiatrists into diplomates, fellows, and associates. Diplomates refer to board-certified psychiatrists. Fellows refer to board-certified psychiatrists who applied for PPA fellow status—a form of membership—which has no bearing on clinical practice. An associate is a member of the PPA who has finished training but has not been board certified. Fellows comprised the majority with 169 psychiatrists (41.3%), and associates only numbered 22 psychiatrists (5%). A female predominance (68.7%) was maintained across all PPA membership types.

Sixty-six psychiatrists (16.1%) in the PhilHealth list were not found in the PPA online directory. Among those listed in the PPA directory, only 44% were accredited with PhilHealth at the time of writing. Psychiatrists classified as diplomates had the highest percentage of accreditation at 51.9%, and regular members had the lowest percentage at 8%. Overall, only 53% of all psychiatrists identified were accredited with PhilHealth at the time of writing.

### 3.2. Exploratory Analysis on Regional Distribution

**Figure 3:**
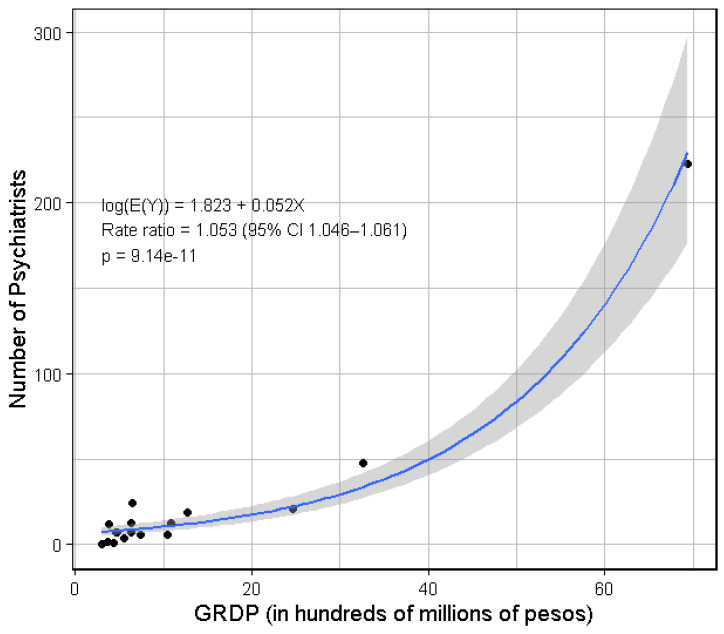
Quasi-Poisson regression plot between 2024 gross regional domestic product (GRDP) with base 2018 prices in hundreds of millions of pesos, and number of practicing psychiatrists. Note: A quasi-Poisson regression was used to account for overdispersion in the Poisson model (dispersion = 4.24). GRDP data was taken from the PSA website [19].

A quasi-Poisson regression indicated that GRDP was strongly positively associated with the number of psychiatrists (Figure 3). For every additional 100 million pesos in GRDP, the expected number of psychiatrists increased by approximately 5.3% (*p* < 0.001; 95% CI 4.6–6.1%). Consistent with this finding, Spearman’s rank-order correlation indicated a positive association (ρ = 0.781; *p* < 0.001).

**Figure 4:**
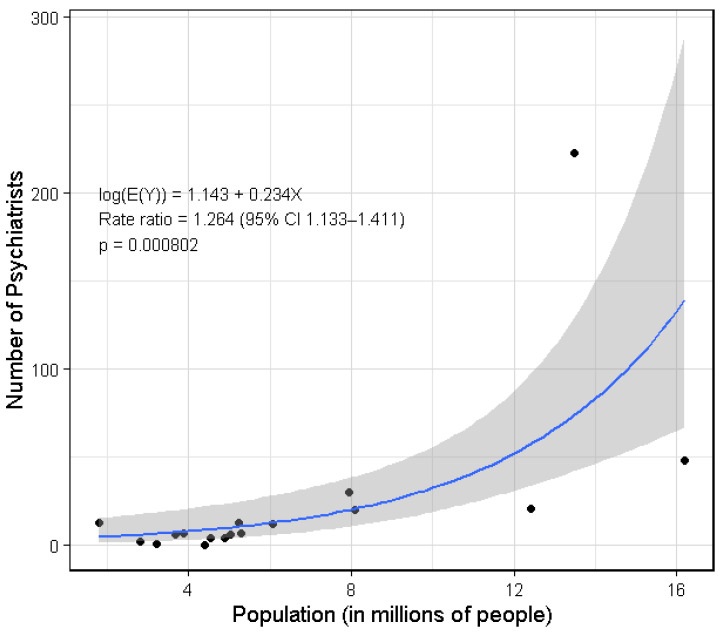
Quasi-Poisson regression plot between number of psychiatrists per population per region (2020 PSA census data) [18]. Note: A quasi-Poisson regression was used to account for overdispersion in the Poisson model (dispersion = 28.67). Number of psychiatrists data comes from data collected from the PPA website in 2025; however, population data comes from the 2020 census taken from the PSA.

A quasi-Poisson regression indicated a significant positive association between population size and number of psychiatrists (Figure 4). Each additional one million population was associated with a 26.4% increase in the expected number of psychiatrists (*p* < 0.001; 95% CI 13.3–41.1%). Consistent with this finding, Spearman’s rank-order correlation indicated a positive association (ρ = 0.753; *p* < 0.001).

**Figure 5:**
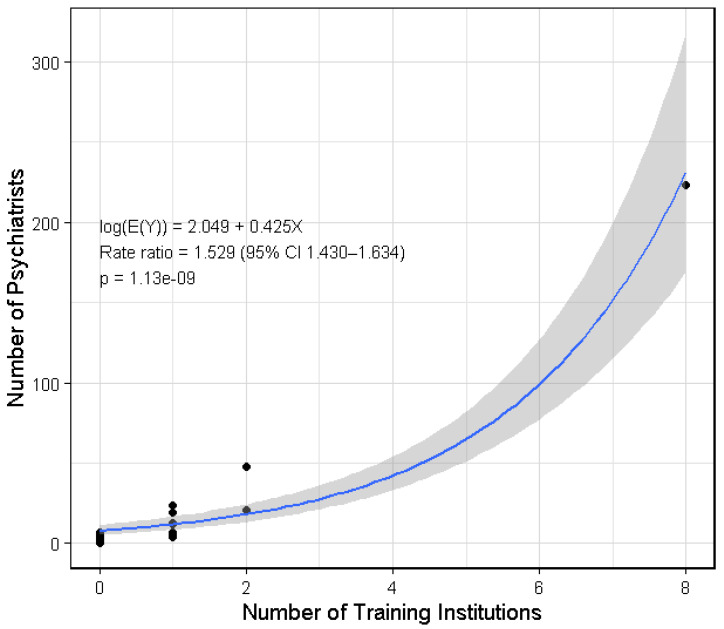
Quasi-Poisson regression plot between number of training institutions and number of psychiatrists by region. Note: A quasi-Poisson regression was used to account for overdispersion in the Poisson model (dispersion = 6.08). Locations of training institutions were taken from the PPA website and tallied per region [20].

Regarding the potential relationship between the number of training institutions and the number of psychiatrists, the PPA website lists 20 training institutions distributed among 11 regions: eight in NCR, one in CAR, one in Cagayan Valley, two in Central Luzon, two in Calabarzon, one in Bicol Region, one in Western Visayas, one in Central Visayas, one in Eastern Visayas, one in Davao Region, and one in the Zamboanga Peninsula. As shown in Figure 5, a quasi-Poisson regression showed a strong positive association between number of institutions and number of psychiatrists. Each additional institution was associated with a 52.9% increase in the expected number of psychiatrists (*p* < 0.001; 95% CI 42–63.4%). Consistent with this, Spearman’s rho showed a significant positive association (ρ = 0.799; *p* < 0.001).

No statistically significant association between membership type and region of practice was found on the chi-squared test of independence: χ^2^(80) = 87.479, *p* = 0.266. Monte Carlo simulation (100,000 replicates) produced a similar result: simulated *p* = 0.268, with a standard error of 0.001 and a 95% confidence interval of [0.266, 0.271], indicating that the observed association is consistent with chance variation.

Likewise, no statistically significant association was found between PhilHealth accreditation and region via chi-squared test: χ^2^(16) = 16.543, *p* = 0.416. Monte Carlo simulation (100,000 replicates) produced a similar result: simulated *p* = 0.426, with a standard error of 0.002 and a 95% confidence interval of [0.419, 0.425], indicating that the observed association is consistent with chance variation.

## 4. Discussion

### 4.1. Psychiatrist to Population Ratio

The Philippines currently has around 409 psychiatrists (listed on the PPA website or in the PhilHealth list of accredited psychiatrists) equating to 0.38 psychiatrists per 100,000 population, a notable decrease from the 548 psychiatrists (0.5 per 100,000) reported by the WHO in 2020 [9]. Both are much lower than the WHO’s recommended minimum of 10:100,000 [24]. This minimum assumes task-sharing with primary care physicians. The extent to which this task-sharing is practiced in the country is unknown at present. Notably, 53.48% of psychiatrists practice in the NCR, making the situation more dire outside of the capital.

For comparison, the WHO reported that globally, on average there are 0.3 psychiatrists per 100,000 population [25]. Based on 2016 data [26], the WHO reported that among Southeast Asian nations, Singapore had the greatest number of psychiatrists (4.91:100,000), followed by Malaysia (1.05:100,000), then by Thailand (0.72:100,000), followed by the Philippines (0.52:100,000). The rest of Southeast Asia had around 0.3 or fewer psychiatrists per 100,000. Norway had the greatest reported number (46:100,000), while many African countries had much less than 0.1:100,000.

The country’s ratio seems to have decreased since 2016, likely due to population growth outpacing the increase in the number of psychiatrists. Another possibility is outmigration [27], as three psychiatrists listed in the PPA declared practice in foreign countries. In addition, other psychiatrists who may have left the country may not be listed in the PPA. Lastly, PPA and PhilHealth online-accessible registries may not be completely updated.

### 4.2. Psychiatrist Geographical Distribution

All three pairs of quasi-Poisson regression models and Spearman’s rank-order correlation indicated statistically significant positive associations between the predictors (GRDP, population, and number of training institutions) and number of psychiatrists. Emphasizing that this was primarily an exploratory analysis, these results should be interpreted cautiously. While it would appear that psychiatrists tend to be more numerous in areas with higher GRDP, populations, and training institutions, it is important to recognize that the relationship may not be causal. It is also important to note that the predictors may influence each other, i.e., more institutions may be found in regions with greater economic prosperity, greater populations may be associated with higher GRDP, population itself may be a scaling factor that increases the value of all variables, etc. Nevertheless, these results indicate that a positive association exists between these variables which may be discussed.

Chi-squared tests for independence, supplemented with Monte Carlo simulations, found no significant association between region of practice, PhilHealth accreditation, and PPA membership type. This may reflect a true lack of association or limited statistical power due to the small sample size; hence, these results should be interpreted with caution.

Apart from random variability, and beyond regional metrics, other more granular factors may have influenced psychiatrist distribution, e.g., the presence of geographically isolated and disadvantaged areas, socioeconomic factors such as livelihood of the regional populace, and local culture may be important considerations. Regional healthcare infrastructure, local government and workforce policies, peace and order, also influence psychiatrist distribution. Additionally, the relationship between psychiatrist density and regional factors might not be strictly linear, pointing to the need for more nuanced approaches in future research.

Taken together, the data suggests that psychiatrists are more likely to concentrate in urban centers with higher GRDP and the presence of training institutions (Figure 3 and Figure 5). Notably, as shown in Table 1 which lists psychiatrist-to-population ratio, the top 10 regions with the best ratios all have training institutions. Several factors may contribute to this concentration pattern. Urban areas may serve as population hubs with higher patient volumes, a higher standard of living, and a greater capacity to pay professional fees, as suggested by GRDP data. The presence of training institutions likewise requires the availability of consultant psychiatrists as both trainers and supervisors to trainee psychiatrists.

Conversely, rural areas and less developed regions with lower psychiatrist density may face challenges, particularly delays in diagnosis and treatment, and logistical barriers related to travel and scheduling. The disparity is aggravated by the fact that psychiatrists may not always relocate to regions with the highest population, as shown in Table 1 and the outlier in Figure 4.

### 4.3. Gender Distribution of Psychiatrists

There were more female than male psychiatrists. An in-depth discussion on this is beyond the scope of this paper, but to a minority of patients, patient sex and the nature of psychological disease may influence preference of therapist’s sex, e.g., masculine men may prefer masculine therapists, and severely depressed men may prefer a female therapist, while some women prefer female therapists for a variety of reasons such as comfort of self-disclosure [28,29,30,31]. Accounting for differences in culture, future local studies may assess if the same is true for the Philippines.

### 4.4. PhilHealth Accreditation

Regarding PhilHealth accreditation, MH conditions were not covered by PhilHealth until 2010, when inpatient coverage for psychiatric conditions was eventually included [32]. With the lack of, and eventually limited, coverage for mental health, many psychiatrists did not register to become accredited PhilHealth providers. In 2023, the PhilHealth Outpatient Benefits Package for Mental Health was officially launched following Circular 2023-0018, allowing for coverage of initial assessments, follow-up consultations, diagnostic tests, and psychosocial support [33]. General coverage can now be supplied by accredited mental health providers.

The low accreditation rates among psychiatrists raise concerns regarding achieving the goals of the package. Patients will have difficulty finding accredited providers, likely face longer waiting times, and may have to travel farther to avail themselves of the package. As in the past, patients may be disincentivized from seeking care entirely or instead look to free government mental health services, which are mostly in urban centers and have long wait times. Otherwise, they will have to continue their care with non-accredited providers out-of-pocket. It should be noted that no formal studies or statistics have been released on the implementation and utilization of the Philhealth Outpatient Package.

Incentives and efforts to improve accreditation rates should be considered to increase the number of accredited psychiatrists. Accredited providers will be able to provide patients with mental health services with significantly less out-of-pocket expenditure, with PhilHealth coverage of up to PHP 9000 for general services and PHP 16,000 for specialty services annually [33].

Aside from accreditation, mental health service providers should also be made aware of the referral pathways stipulated by the Outpatient Package, including specialty services only available in higher level institutions.

### 4.5. Engaging a Qualified Psychiatrist

In areas lacking psychiatrists, the roles of emergency and non-emergency psychiatric care provision fall on primary care physicians, who might not be trained sufficiently to identify nuanced cases. The selection of treatments in emergent cases likewise may be limited, with basic treatment options such as antidepressants, antipsychotics, or other psychotropics being unavailable. Outside of the NCR, patients may also be referred to locations far from their residence.

For non-emergency care, patients may seek psychiatric consultation through recommendations from their social circle, or by searching online for psychiatrists practicing in their area or those offering telemedicine services. Caution and due diligence must then be exercised by the patient to verify the credibility of the psychiatrist, as there have been news reports of individuals impersonating physicians, with at least one who has claimed to be a psychiatrist [34,35,36]. This highlights the importance of credible sources, e.g., the PPA directory and the PhilHealth list of accredited psychiatrists. While the actual number of psychiatrists practicing in the Philippines may be more, the Internet-surfing public might only be able to encounter the psychiatrists with an online presence, highlighting the significance of analyzing data that is freely available online.

The PPA confers the title of diplomate to those who have passed the psychiatry board exam. This certification is required by many tertiary hospitals to practice psychiatry. A diplomate may apply to become a fellow of the PPA for benefits, such as professional development. Regular PPA members are active trainees, and Associates are those who have completed training but have not yet passed the board exams. Regular members and Associates may call themselves psychiatrists informally but cannot identify themselves as board certified.

While no official guidelines exist to determine which physician can or cannot practice psychiatry, it is generally accepted that complicated psychiatric cases and intensive evaluation and management are within the purview only of those who are certified specialists. As such, although these physicians have completed training, they may be limited to outpatient or less complicated cases until they have acquired certification. This has implications for interpreting the number of psychiatrists granted the apparent stratification of qualifications.

There may also be cases where some non-psychiatrist physicians are practicing as outpatient psychiatrists. The Philippine Medical Association code of ethics indicates that a physician has the right to choose patients [37], and there may indeed be general practitioners exercising this right by selectively treating psychiatric patients. To some extent, this may be permissible if they do not claim to be certified psychiatrists and refer specialist-requiring patients accordingly. It is true that psychiatric care should not be gate-kept, and both patients and psychiatrists will benefit from general practitioners bearing part of the burden. However, the guiding principle must be to provide care only within the realm of one’s knowledge and expertise, erring on the side of caution.

### 4.6. Relation to Telemedicine in the Context of Post-Pandemic Psychiatric Practice

There has been a remarkable increase in psychiatric burden in the country, and this has been especially true during the COVID-19 pandemic. International reports indicate that waiting times to be seen by a MH professional had reached several months [38,39]. Not only had the pandemic and lockdowns caused stress and MH perturbation, but they had also limited the options for accessing support [40], thus pushing psychiatric practice to transition to telemedicine.

As the pandemic subsided, many psychiatrists and MH professionals opted to maintain their practice of telepsychiatry, with some preferring this over in-person consultation, owing to perceived acceptability, appropriateness, feasibility of the technology, and improved access [41,42]. There is also evidence that telepsychiatry has remained a significant modality of psychiatric care provision abroad post-pandemic [43,44].

Telemedicine helps address many aspects of urban-rural disparity by providing a means to bypass geographical constraints. In terms of effectiveness in achieving desired mental health outcomes, telemedicine in psychiatry has been associated with good patient satisfaction, reliable diagnoses, and good clinical outcomes [45]. However, it should be noted that those in rural areas may also face difficulties due to limitations in infrastructure, namely poor Internet connectivity and reception, lack of knowledge of the existence of these services, technological literacy, hesitancy to use telemedicine, and access to devices [41,46]. Scheduling is practitioner-dependent regardless of mode of consultation, but in the case of telemedicine, there may be the added factor of technological literacy. On the other hand, telepsychiatry consults are more likely to be completed and followed-up compared to in-person consults [43]. There is no available Philippine data regarding likelihood of follow-up and consult completion, but it may be hypothesized that due to ease of use, the experience in the Philippines may be similar.

It should be noted that no standardized training for telepsychiatry in the Philippines exists at present, which may be associated with discrepancies in implementation and provision of care. Regardless, telepsychiatry is likely to persist in the future, and may be leveraged to address geographical barriers, while physical availability and accessibility catches up.

### 4.7. Socioeconomic Factors Affecting Psychiatric Consultation

A systematic analysis from 2020 analyzed studies tackling mental health help-seeking barriers and facilitators for Filipinos living locally and overseas [47]. It was noted that the rate of formal psychological help-seeking of local Filipinos was generally low at 22.19%, indicating that help from family and friends or lay networks are prioritized, and professional help is sought only as a last resort. Filipinos seek professional help usually in combination with other sources of care such as from family and friends, or only when the mental health problem is perceived as severe. Interestingly in the analysis, the authors noted a positive attitude towards help-seeking among users of online counseling, but Filipinos in crisis centers still preferred receiving help from religious clergy or family members, with mental health units as the least preferred setting in receiving help. The researchers also noted in their review that Filipino women refrained from disclosing their problems to others. Prior negative experiences with a psychological health professional were also determined to be a common barrier for subsequent help-seeking.

In the same systematic analysis, the authors noted the most commonly identified classes of barriers to mental health help-seeking [47]. These barriers were financial constraints (e.g., lack of insurance, high costs), self-stigma (e.g., fear of negative judgment, shame, being labeled as ‘crazy’, self-blame), and social stigma (e.g., wish to avoid putting family reputation at stake). Conversely, the authors also identified notable facilitators for help-seeking common to both local and overseas Filipinos, namely severity (i.e., more severely perceived problems prompted help-seeking), social influence (e.g., encouragement from family and friends, witnessing friends seeking help), and financial capacity.

The pattern of Filipino help-seeking seems primarily driven by the lack of funds as well as general culture. However, these are not unchangeable since the identified facilitators are the exact or near opposites of the identified barriers. In relation to the present study, financial disparities in the Philippines are known and recognized throughout the country, and thus for these patients, psychiatric help-seeking does not seem like a viable option.

Given that there is some positive attitude towards online counseling, Filipino psychiatrists perhaps should consider launching their own online practices alongside their in-person practices. This may be helpful for patients who prefer the safety and familiarity of their homes rather than the potentially unfamiliar venue of a clinical office while experiencing the difficulty of their mental health concerns.

Also, given that Filipinos prefer lay help, lay people, particularly those helpful and close to the patient, can and should be recognized as allies in the management of mental health disorders. One potential intervention that the limited number of psychiatrists may do is strengthen education drives to dispel stigma, empower lay people in aiding each other, and to allow lay persons to identify cases which must be seen by a psychiatrist.

### 4.8. Factor of Costs of Management

In the case where patients do follow through and seek consultation with psychiatrists, there is a chance that they will be prescribed medications to manage their condition alongside psychotherapy and other nonpharmacologic managements.

A 2021 study surveyed global use of psychotropics and identified the Philippines as the country with the lowest consumption of psychotropics at 0.93 Defined Daily Dose (DDD) per 1000 inhabitants per day, followed by Venezuela at 3.69 DDD per 1000 inhabitants per day [48]. The WHO defines DDD as the assumed average maintenance dose per day for a drug used for its main indication in adults [49]. This may be a proxy indicator that Filipinos generally do not spend much on psychiatric medication, most likely due to financial constraints. This is one other potentiating factor for low help-seeking behavior, i.e., prohibitive prices for pharmacologic management may preclude follow-up.

The problem of high psychiatric medication costs is complex, and long-term solutions should be explored through further research. Granted that these obstacles are not easy to overcome, this could be a signal for psychiatrists to further utilize social workers and maximize nonpharmacologic therapy. This is also a signal for psychiatrists to become accredited with PhilHealth and to help patients in accessing government-provided financial aid for medications where available, such as the Department of Health Medication Access Program for Mental Health [4].

### 4.9. Strengths and Limitations of the Study

This is a first attempt to describe the distribution of psychiatrists in the Philippines per region. The study collates information freely available from the web. Thus, while there might be more psychiatrists than what is listed on the PPA website, the number of psychiatrists identified here reflect what a potential patient may encounter when seeking out a psychiatrist via the Internet. The paper also attempted to find relationships between declared regions of practice and several variables such as Philhealth accreditation and PPA membership, and regional factors such as GRDP, number of training institutions, and population.

There are several limitations to the study. Other variables were not collected, e.g., psychiatrist age and years in practice, as these were not available from web sources. There are potential gaps in the data from the PPA and PhilHealth databases, and a need for qualitative insights into psychiatrist preferences for practice location. Sex of the psychiatrist could not be verified and was assumed based on traditional naming conventions. Temporal mismatch between data sources precluded combined multivariate analysis.

## 5. Conclusions

The findings of this study highlight the shortage and uneven distribution of psychiatrists in the Philippines which complicates efforts to address the increasing prevalence of MH disorders. The maldistribution—which shows a concentration in urban areas, particularly the NCR—highlights the inequality of service provision across the archipelago. The advent of telepsychiatry and telepsychology presents an opportunity to address both the shortage and uneven distribution. Psychiatrists may also strengthen education drives to recruit and empower lay people for the management of mental health concerns.

The low PhilHealth accreditation rates of psychiatrists is another challenge that needs to be addressed. However, this may be improved with the recent introduction of outpatient mental health benefits by PhilHealth.

Further recommendations to address the unequal distribution include incentivizing practice and improving access to telemedicine. Legislators may explore long-term solutions, such as increasing the budget for mental health training both for specialists and primary care physicians, as well as exploring the possibility of developing training institutions in poorly served locales.

## Figures and Tables

**Table 1:** Number of psychiatrists, population, and psychiatrist to population per region in the Philippines arranged from lowest to highest psychiatrist to population.

Region	Population	Psych:Pop	Psych per 100,000
Bangsamoro (BARMM)	4,404,288	-	0
Southwestern Tagalog Region (Mimaropa)	3,228,558	1:3,228,558	0.031
Caraga (Region XIII)	2,804,788	1:1,402,394	0.071
Soccsksargen (Region XII)	4,901,486	1:1,225,372	0.082
Eastern Visayas (Region VIII)	4,547,150	1:1,136,788	0.088
Northern Mindanao (Region X)	5,022,768	1:837,128	0.119
Ilocos Region (Region I)	5,301,139	1:757,306	0.132
Cagayan Valley (Region II)	3,685,744	1:614,291	0.163
Central Luzon (Region III)	12,422,172	1:591,532	0.169
Zamboanga Peninsula (Region IX)	3,875,576	1:553,654	0.181
Bicol Region (Region V)	6,082,165	1:506,847	0.197
Central Visayas (Region VII)	8,081,988	1:404,099	0.247
Davao Region (Region XI)	5,243,536	1:403,349	0.248
Calabarzon (Region IV-A)	16,195,042	1:337,397	0.296
Western Visayas (Region VI)	7,954,723	1:265,157	0.377
Cordillera Administrative Region (CAR)	1,797,660	1:138,282	0.723
National Capital Region (NCR)	13,484,462	1:60,468	1.654

Note: Data from the 2020 census was taken from the PSA website [18]. Psychiatrists practicing in the Negros Island Region (NIR) were redistributed to either Western Visayas or Central Visayas depending on their declared city or province because the NIR had not yet been formed in 2020. Psych = Psychiatrists; Pop = Population.

## Data Availability

The data used in this study are publicly available, without restrictions, from (1) Philippine Psychiatric Association (PPA) web directory of board-certified psychiatrists (https://philippinepsychiatricassociation.org/psychiatrist/ (accessed on 12 February 2025)), (2) PhilHealth web database of accredited psychiatrists (https://www.philhealth.gov.ph/partners/providers/professional/accredited/ (accessed on 12 February 2025)), and (3) Philippine Statistics Authority (PSA) mid-year population data (https://psa.gov.ph/statistics/population-and-housing/node/164811 (accessed on 12 February 2025)).

## Abbreviations

The following abbreviations are used in this manuscript:

PPA	Philippine Psychiatric Association
PhilHealth	Philippine Health Insurance Corporation
GDP	Gross Domestic Product
UHC	Universal Health Care
MH	Mental Health
WHO	World Health Organization
PSA	Philippine Statistics Authority
GRDP	Gross Regional Domestic Product
NIR	Negros Island Region
NCR	National Capital Region
DDD	Defined Daily Dose
